# Immunogenicity without Efficacy of an Adenoviral Tuberculosis Vaccine in a Stringent Mouse Model for Immunotherapy during Treatment

**DOI:** 10.1371/journal.pone.0127907

**Published:** 2015-05-21

**Authors:** S. Anisah Alyahya, Scott T. Nolan, Cara M. R. Smith, William R. Bishai, Jerald Sadoff, Gyanu Lamichhane

**Affiliations:** 1 Crucell Holland B.V., Janssen Infectious Diseases and Vaccines, Leiden, The Netherlands; 2 Center for Tuberculosis Research, Department of Medicine, Johns Hopkins University School of Medicine, Baltimore, Maryland, United States of America; 3 HHMI, Johns Hopkins University School of Medicine, Baltimore, Maryland, United States of America; Federal University of São Paulo, BRAZIL

## Abstract

To investigate if bacterial persistence during TB drug treatment could be overcome by modulation of host immunity, we adapted a clinically-relevant model developed for the evaluation of new drugs and examined if immunotherapy with two adenoviral vaccines, Ad35-TBS (AERAS-402) and Ad26-TBS, could shorten therapy in mice. Even though immunotherapy resulted in strong splenic IFN-γ responses, no effect on bacterial replication in the lungs was seen. Multiplex assay analysis of lung samples revealed the absence of cytokine augmentation such as IFN-γ, TNF-α and IL-2, suggesting that immunization failed to induce immunity in the lungs. In this model, we show that IFN-γ levels were not associated with protection against disease relapse. The results obtained from our study raise questions regarding the traits of protective TB immunity that are relevant for the development of future immunotherapeutic and post-exposure vaccination strategies.

## Introduction

For a curable disease, Tuberculosis (TB) continues to take the lives of unacceptably large numbers of individuals worldwide. One of the biggest obstacles to eradicating TB is the long duration of treatment with a complicated regimen that lasts over a period of 6 months. In communities with poor healthcare infrastructure, interruption of therapy is frequent leading to the rise of multi-drug tuberculosis (MDR-TB). Treating MDR-TB is far more complex and many times more expensive than standard therapy [1]. Thus one solution to this predicament would be the development of shorter effective therapies.

Despite recent breakthroughs in drug therapy, one issue that limits the ability of drug regimens to significantly shorten therapy duration is the presence of a subpopulation of *M*. *tuberculosis* (*M*. *tb*) bacilli that are temporarily tolerant and persist despite drug action. The exact mechanism of persistence is unclear, but it involves the presence of bacteria that are slow-growing and often non-culturable. Furthermore, due to suboptimal immunity following chronic infection, the host immune response appears unable to compensate where drug therapy fails and act upon the persister population [2–4]. Nonetheless, therapy with drugs that target different bacterial growth mechanisms will eventually eliminate the majority of bacteria and cure the host, following prolonged treatment.

Although new, faster-acting drugs are in development, history predicts that their very usage (and especially over and improper usage) may lead to selection of resistant *M*.*tb* strains. Therefore, it is necessary to consider alternative strategies in parallel such as modulation of host immune responses to target these persister cells. However, one of the inherent challenges of boosting host immunity in the presence of *M*.*tb* is the development of detrimental exaggerated inflammatory responses, known as Koch’s phenomenon. As a consequence, the development of immunotherapeutic or post-exposure vaccines stalled following Robert Koch’s introduction of tuberculin therapy where numerous lethal complications were eventually seen [5]. In spite of this, the rise of MDR-TB in recent years has reinvigorated interest in the pursuit of antibiotic-independent strategies to combat *M*.*tb* and has resulted in the development of new TB experimental immunotherapies [6].

Besides immune modulation as a way to shorten drug treatment, post-exposure vaccines that aim to prevent latent TB from developing into active TB must also be able to target the persister population. The importance of developing post-exposure vaccines is reinforced by the challenges of developing prophylactic vaccination that is more effective than BCG due to the immaturity of cell-mediated immunity in infants unexposed to TB [7]. Thus, a post-exposure vaccine that targets adults with latent TB is considered to be a more realistic goal in the quest to reduce the worldwide burden of TB [8]. Although the nature of persistent organisms in latent TB may differ from persisters that arise during active TB therapy, there may also be similarities that could be targeted by both an immunotherapeutic and a post-exposure vaccine. In particular, the notion that non-replicating dormant bacteria are periodically replicating is supported by the ability of isoniazid, a drug that targets replicating bacteria to treat latent TB [9]. Thus, generating an immune response that could continuously eliminate bacteria that periodically leave the non-replicating state would be one of the goals of both immunotherapy and post-exposure vaccination.

With this in mind, we designed a study to investigate whether boosting TB-infected, drug-treated mice, with a heterologous prime-boost regimen of two different adenoviral vaccines containing specific TB antigens could enhance the immune response against *M*.*tb* and consequently shorten chemotherapy. The non-replicating adenoviral vaccine Ad35-TBS (also known as AERAS-402), expressing Ag85A, Ag85B and TB10.4 has previously demonstrated protection against infection in a prophylactic mouse model of TB and is currently one of the most advanced new TB vaccines in clinical development [10,11]. Due to the elicitation of vector-induced immunity limiting the usage of multiple booster vaccinations, the Ad26-TBS vaccine containing the same TB antigens was developed to bypass this issue, allowing for heterologous boosting of the Ad35-TBS vaccine (manuscript in preparation).

Here, we report that, immunization with these vectored vaccines successfully induced robust immune responses without eliciting pathology but failed to control bacterial growth at every time point measured. We further identified a potential mechanism of failure by analysing measurements of lung cytokine levels which revealed a discrepancy between splenic immune responses to that found at the site of local infection. The results of our study raise many relevant questions for TB vaccine development regarding the traits of protective TB immune responses as well as our understanding on the nature of persisters.

## Results

### Study design

In order to study the effects of vaccination on shortening standard TB treatment, we modified a murine model developed for the evaluation of new TB drugs for the first time in the context of immunotherapy. The original drug-treatment model is based on the measurement of culture-positive relapse, which is defined as the isolation of 1 or greater colony forming units (CFU) after plating the entire lung homogenate three months after the completion of therapy [12,13]. The addition or substitution of new TB drugs with a superior sterilizing effect compared to the standard six month therapy have been shown to reduce the relapse proportions when treatment was shortened to four months or less [12,14]. Based on this, we hypothesized that if Ad35-TBS and Ad26-TBS could induce sterilizing immune responses, the relapse proportion in the immunized group would be lower than that of the control treatment group.

**Fig 1:**
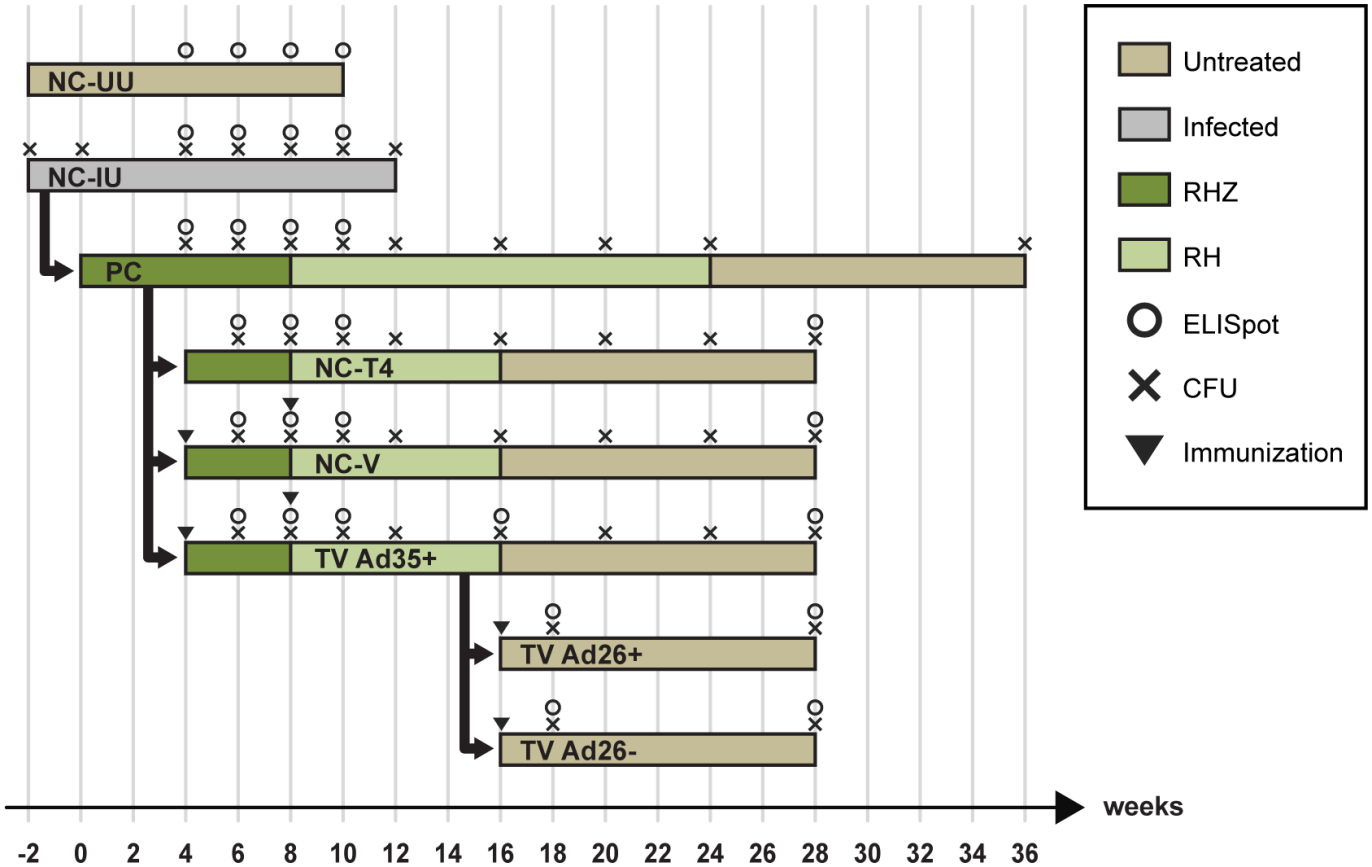
Schematic of study design. 420 mice were divided into animals that were uninfected (NC-UU) (20 mice) and those that were infected with *M*.*tb* at week -2 (NC-IU) (400 mice). At week 0, 365 mice from NC-IU were removed to create the PC cohort that was initiated on therapy with R, H and Z via oral gavage, 7 days per week. The remaining NC-IU animals were left untreated up to week 12, when they became moribund and were sacrificed. At week 4, 284 animals from the PC cohort were randomized into groups NC-T4, NC-V and TV Ad35+. At this time point, mice in the NC-V and TV Ad35+ groups received intramuscular immunotherapy with 10^10^ viral particles of Ad35 empty vector or Ad35-TBS vaccine, which were respectively boosted at week 8. Animals in the PC, NC-T4, NC-V and TV Ad35+ groups were switched to therapy with R and H at week 8 which lasted to week 16, except for the PC group which received therapy up to week 24. At week 16, when therapy was shortened, 56 mice from the TV Ad35+ group were removed and divided into 2 further groups, TV Ad26+ and TV Ad26- which received immunotherapy with Ad26-TBS and Ad26, respectively. All animals were followed through for 3 months following therapy cessation at week 16 and week 24 (PC). Animals were subsequently sacrificed for the final lung CFU enumeration. Multiplex analysis on lung samples were carried out at times indicated for CFU counting, from week 4 onwards.

The main relapse study was designed as depicted in Fig 1, and comprised of eight arms that were randomized at specific time points. The positive control group (PC) were animals that underwent the complete TB therapy over 24 weeks, similar to that in the clinic. Here, 8 weeks of Rifampicin (R), Isoniazid (H) and Pyrazinamide (Z) were delivered in the initiation phase followed by 16 weeks of RH in the continuation phase. In this group, 0–2% of animals are expected to relapse [12]. The main negative control group (NC-T4) were animals that received a shortened continuation phase of 8 weeks which resulted in 16 weeks of total therapy where the established relapse proportions have been found to be around 90% [12]. Animals in the treatment vaccinated arm (TV Ad35+) received two doses of immunization with 10^10^ viral particles of Ad35-TBS at weeks 4 and 8 and underwent a shortened therapy similar to the NC-T4 group. Vaccination timing was chosen at a time when the bacterial burden was known to be reduced considerably by treatment as we were concerned about the induction of exacerbated immune responses in the presence of large numbers of bacteria [15]. In all groups, drugs were administered by oral gavage daily.

To study the effect of vaccination when bacterial numbers became undetectable at week 16, we further divided the TV Ad35+ group into a group that received a third dose of vaccination with a different Adenovirus serotype Ad26-TBS (TV Ad26+). Due to the presence of neutralizing antibodies to the Adenoviral particles, we theorized that a heterologous boost with a different viral serotype would result in further augmentation of the immune response that may target persister cells at this stage. For each vaccination group, we added a matching control group (TV Ad26- and NC-V) that comprised of Ad26 and Ad35 vectors devoid of TB antigens respectively, to exclude the contribution of non-specific vector-induced immune responses to the final study outcome. Finally, two further arms consisting of animals that were infected but left untreated (NC-IU) or uninfected and untreated (NC-UU) were added to serve as controls for bacterial burden and baseline immune responses.

At various time points throughout the study, animals were sacrificed to determine the number of colony-forming units (CFU), the immune response to vaccination and histopathology (Fig 1). Table 1 illustrates the total number of animals utilized for each data point. Based on the outcomes of our preliminary study and previous published experiments, we determined, that a sample of 23 mice per group for the final relapse time point provided 80% power to detect a difference between 90% relapse rate (in the negative control group, eg. NC-T4) and a 50% relapse rate in the vaccinated group (eg. TV Ad35+) [12]. Furthermore, a sample size of 23 mice per group provided 80% power to detect differences of 0.25 mean (log) CFU counts between the different groups. We did not find smaller differences to be meaningful in terms of shortening the duration of treatment.

**Table 1:** Numbers of mice used in the study at each time point.

Week	PC	TV Ad35+	TV Ad26+	TV Ad26-	NC-V	NC-T4	NC-IU	NC-UU	Total
**-2**							5		**5**
**0**							5		**5**
**4**	5						5	5	**15**
**6**	5	5			5	5	5	5	**30**
**8**	5	5			5	5	5	5	**30**
**10**	5	5			5	5	5	5	**30**
**12**	8	8			8	8	5		**37**
**16**	10	10			10	10			**40**
**18**			5	5					**10**
**20**	10	10			10	10			**40**
**24**	10	10			10	10			**40**
**28**		23	23	23	23	23			**115**
**36**	23								**23**
**Total**	**81**	**76**	**28**	**28**	**76**	**76**	**35**	**20**	**420**

420 female BALBc mice were used to obtain data for immunological analysis, relapse CFU measurements and histopathology. Numbers were gradually increased to ensure sufficient power to detect differences between the groups as described previously. NC-UU = negative control, uninfected, untreated; NC-IU = negative control, infected, untreated; PC = positive control, standard therapy; NC-T4 = negative control, shortened therapy; NC-V = negative control, Ad35 empty vector, shortened therapy; TV Ad35+ = Ad35-TBS immunotherapy, shortened therapy; TV Ad26+ = Ad26-TBS immunotherapy, shortened therapy; TV Ad26- = negative control, Ad26 empty vector, shortened therapy.

### Splenic ELISpot analysis following immunotherapy

**Fig 2:**
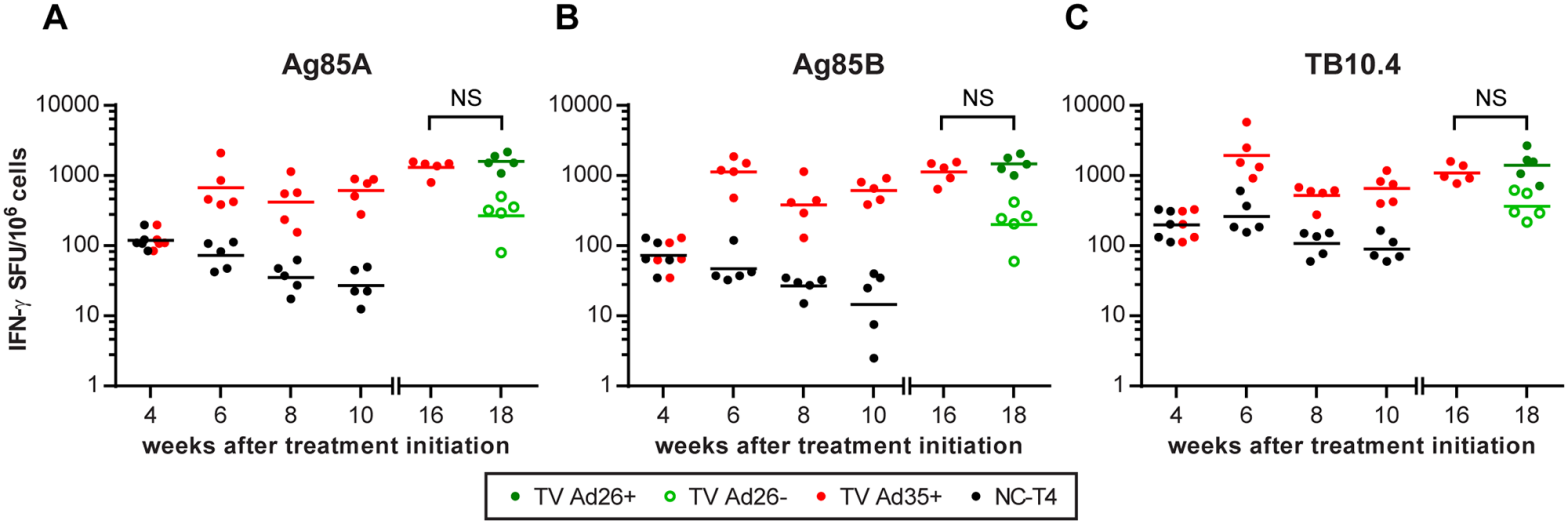
Immunogenicity of Ad35-TBS and Ad26-TBS immunotherapy. At week 4, baseline IFN-γ production to pooled peptides covering the whole sequence of Ag85A, Ag85B and TB10.4 were measured by ELISpot prior to randomization (refer to Fig 1). At this time point, the values between the NC-T4 and TV Ad35+ are identical. Following randomization, animals in the TV Ad35+ group received immunotherapy at weeks 4 and 8 with 10^10^ viral particles of Ad35-TBS intramuscularly. At week 16, baseline immune responses were measured in the TV Ad35+ group prior to randomization into TV Ad26+ and TV Ad26- groups. The TV Ad26+ animals then received heterologous boosting with 10^10^ viral particles of Ad26-TBS at week 16 whilst the TV Ad26- received empty Ad26 vector as control. p-values were calculated two weeks after each immunotherapy (weeks 6 and 10) and are detailed in Table 2 (ANOVA followed by Tukey’s multiple comparisons test, n = 5 at each time point for all groups). Lines depict geometric mean. No significant differences were observed following Ad26-TBS boosting at week 16 for all groups (NS = not significant, t-test). SFU = splenocyte forming units.

In order to determine the immune response to vaccination, ELISpot analysis was conducted immediately prior to and two weeks after vaccinations with Ad35-TBS, Ad26-TBS and their respective empty vector controls (Fig 1). Considering the initial TB infection to be the priming immune response, we observed that immunotherapy with Ad35-TBS at weeks 4 and 8 boosted the immune response to vaccine antigens by week 10 compared to the unvaccinated treated control, NC-T4, as measured by splenocyte IFN-γ production to pooled peptide antigens (Fig 2, Table 2). Heterologous boosting with Ad26-TBS however did not augment the immune response further compared to week 16. Nonetheless, a significant difference to the empty vector control group TV Ad26- was detected, suggesting that boosting maintained the heightened responses achieved by the previous two immunizations with Ad35-TBS (Fig 2, Table 2). Due to the similarity in responses between the control groups, only data from the main control group NC-T4 is presented in Fig 2. Data from all study groups for all time points can be found in S1 Fig.

**Table 2:** Statistical analysis of immunogenicity following immunotherapy. .

Cohort	Peptide	Wk6	Wk10	Wk18
**TV Ad35+ vs NC-T4**	Ag85A CD4	*	*	
	Ag85A CD8	****	****	
	Ag85B CD8	****	*	
	Ag85A pool	****	****	
	Ag85B pool	****	****	
	TB10.4 pool	****	**	
**TV Ad26+ vs TV Ad26-**	Ag85A CD4			*
	Ag85A CD8			***
	Ag85B CD8			*
	Ag85A pool			**
	Ag85B pool			***
	TB10.4 pool			**
**TV Ad35+ vs NC-IU**	Ag85A CD4	NS	NS	
	Ag85A CD8	NS	NS	
	Ag85B CD8	NS	NS	
	Ag85A pool	*	NS	
	Ag85B pool	NS	NS	
	TB10.4 pool	NS	NS	

Results of statistical analysis two weeks following immunotherapy with Ad35-TBS and Ad26-TBS, for all peptides tested in the ELISpot assay.

* p-value <0.05

** p-value <0.01

***p-value <0.001

****p-value <0.0001

One-way ANOVA with Tukey’s multiple comparisons test and t-test (TV Ad26+ vs TV Ad26-) was used for analysis.

NS = not significant.

**Fig 3:**
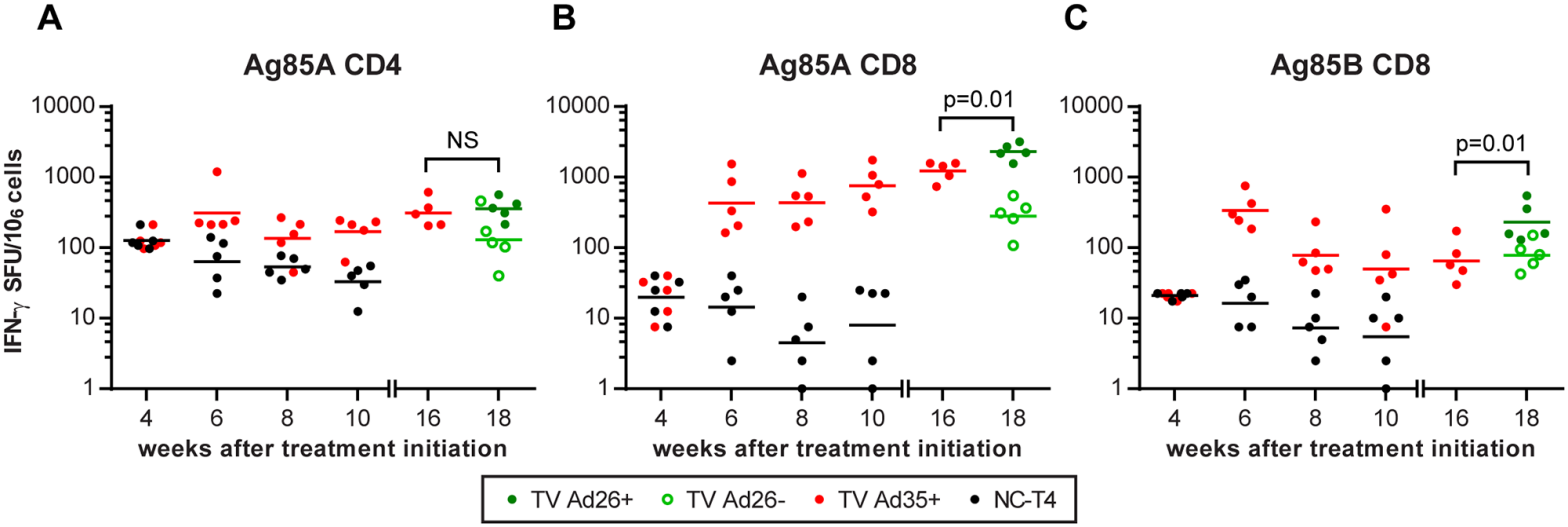
T-cell responses following Ad35-TBS and Ad26-TBS immunotherapy. At week 4, baseline IFN-γ production following stimulation with CD4 and CD8 peptides to Ag85A and CD8 peptides to Ag85B were measured by ELISpot prior to randomization (refer to Fig 1). At week 4, animals in the NC-T4 and TV Ad35+ have not been randomized and are identical to one another. Immediately following randomization at week 4 into the respective groups, immunotherapy was administered via intramuscular injection to the TV Ad35+ animals with 10^10^ viral particles of Ad35-TBS which was boosted at week 8. At week 16, baseline immune responses were measured in the TV Ad35+ group prior to randomization into TV Ad26+ and TV Ad26- groups. The TV Ad26+ animals then received heterologous boosting with 10^10^ viral particles of Ad26-TBS at week 16 whilst the TV Ad26- received empty Ad26 vector as control. p-values were calculated two weeks after each immunotherapy (weeks 6 and 10) and are detailed in Table 2 (n = 5 at each time point for all groups). Lines depict geometric mean. Boosting with Ad26-TBS resulted in significant heightening of CD8 responses to Ag85A (p = 0.01, t-test) and Ag85B (p = 0.01, t-test) at week 18 compared to week 16 that was not observed for CD4 responses to Ag85A (NS = not significant, p = 0.66, t-test). SFU = splenocyte forming units.

As pooled peptide antigens contained peptides that non-specifically stimulated both CD4+, CD8+ as well as other IFN-γ-producing cell types, we used peptide antigens that have been previously identified to be T-cell specific in order to tease out the different T-cell responses (S1 Table). The incomplete repertoire of T-cell epitopes previously discovered for use in BALBc mice prevented us from testing responses to CD4+ and CD8+ T-cells for all antigens equally [10,16]. We therefore focused on Ag85A, a major antigen contained within our vaccines and the MVA85A vaccine, where both the CD4+ and CD8+ epitopes have been identified and characterized [10,16,17]. We observed that two doses of immunotherapy with Ad35-TBS induced significant CD4+ responses to Ag85A by week 10 when compared to the NC-T4 control (p = 0.03, ANOVA) (Fig 3a, Table 2). However, heterologous boosting with Ad26-TBS at week 16 only maintained the CD4+ response at week 18 without a significant enhancement from baseline levels measured at week 16 (Fig 3a). In contrast, compared to the NC-T4 control cohort which exhibited lower overall CD8+ responses, immunotherapy with both Ad35-TBS and Ad26-TBS was able to induce highly increased CD8+ IFN-γ secretion to Ag85A by week 10 (TV Ad35+: p<0.0001, ANOVA) which was further boosted by Ad26-TBS at week 18 (Fig 3b,Table 2). We also tested responses to another CD8+ peptide to Ag85B that have been previously identified to compare it with the CD8+ responses to Ag85A [10,16]. Two weeks after the first immunotherapy with Ad35-TBS, the CD8+ response to Ag85B was elevated (p<0.0001, ANOVA) but unlike Ag85A, the response diminished by week 10, when the differences between vaccinated and unvaccinated NC-T4 animals remained statistically significant but less marked (p<0.05, ANOVA). (Fig 3c, Table 2). However, boosting with Ad26-TBS increased the CD8+ responses to Ag85B compared to pre-boosting levels at week 16 (Fig 3c).

**Fig 4:**
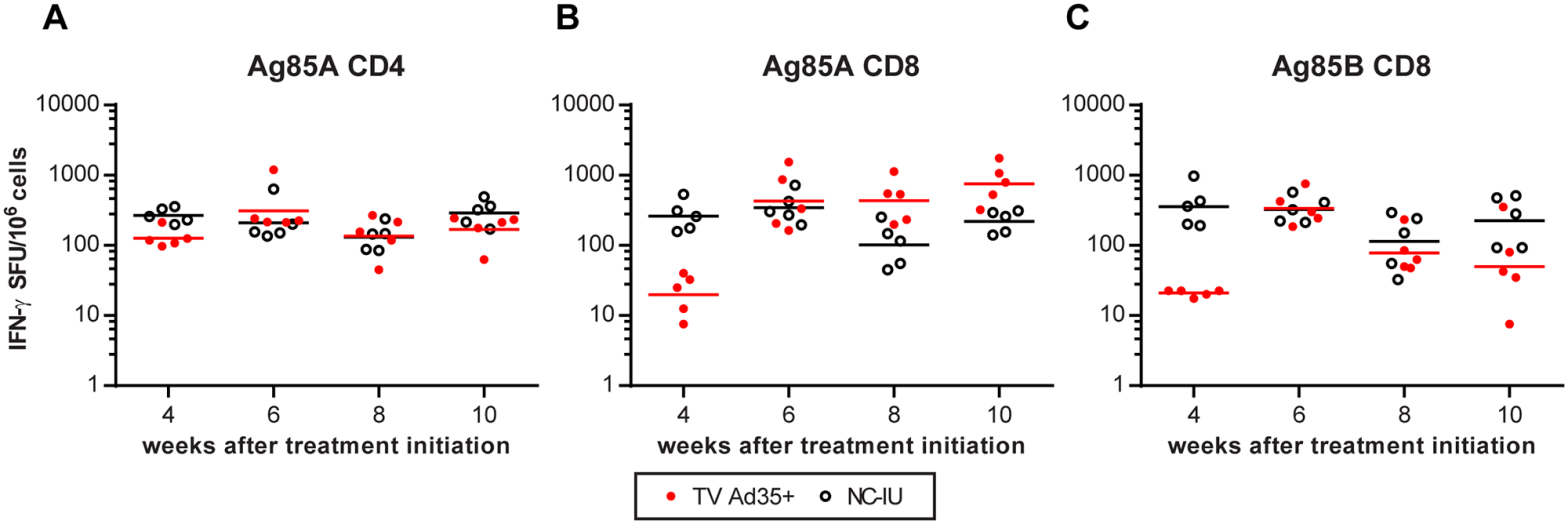
Comparison of T-cell responses between animals receiving immunotherapy and chronically infected untreated animals. ELISpot assay was conducted to measure IFN-γ production in the TV Ad35+ and NC-IU animals following stimulation with A) CD4 peptide antigen to Ag85A B) CD8 peptide antigens to Ag85A and C) CD8 peptide antigens to Ag85B. Mice in the TV Ad35+ group received immunotherapy via intramuscular injection with 10^10^ viral particles of Ad35-TBS at weeks 4 and 8. Lines depict geometric mean and p-values were calculated using ANOVA followed by Tukey’s multiple comparisons test (n = 5 at each time point for all groups) and are detailed in Table 2. SFU = splenocyte forming units.

Previous studies have demonstrated T-cells to be in a state of terminal differentiation and progressive impairment in chronic TB infection [2–4,18]. On the contrary, we observed persistently heightened CD4+ immune responses to Ag85A in infected untreated animals (NC-IU) which was not significantly different from animals receiving two doses of Ad35-TBS immunotherapy (Fig 4a, Table 2). Of note, CD8+ IFN-γ secretion was visibly lower for TV Ad35+ group compared to the NC-IU group at week 4 prior to immunization, reflecting the difference in bacterial burden due to therapy in the TV Ad35+ group (see below for bacterial count). However, the numbers of IFN-γ secreting CD8+ T-cells increased substantially following the first immunization at week 4 and this response was maintained after the second immunization at week 8. Although there appears to be a trend towards higher CD8 Ag85A responses in the TV Ad35+ group compared to the NC-IU group at week 10, the differences were not found to be statistically significant (p = 0.27, ANOVA) (Fig 4b, Table 2). The CD8+ response to Ag85B followed different dynamics, as initial immunization raised the CD8+ response by week 6, but this response was insufficient in overcoming the maximal CD8+ response reached in the infected untreated animals. This observation can be seen more clearly in a separate experiment we conducted where Ag85A CD8 responses surpassed levels measured in the infected untreated animals whereas the Ag85B response remained indistinguishable from the infected untreated controls (S2 Fig). These results imply that immunotherapy with Ad35-TBS augmented specific CD8+ IFN-γ responses to Ag85A but to a lesser degree for Ag85B, beyond that which was possible in a natural state of infection.

### Multiplex enzyme-linked immunosorbent assay for lung cytokine responses

**Fig 5:**
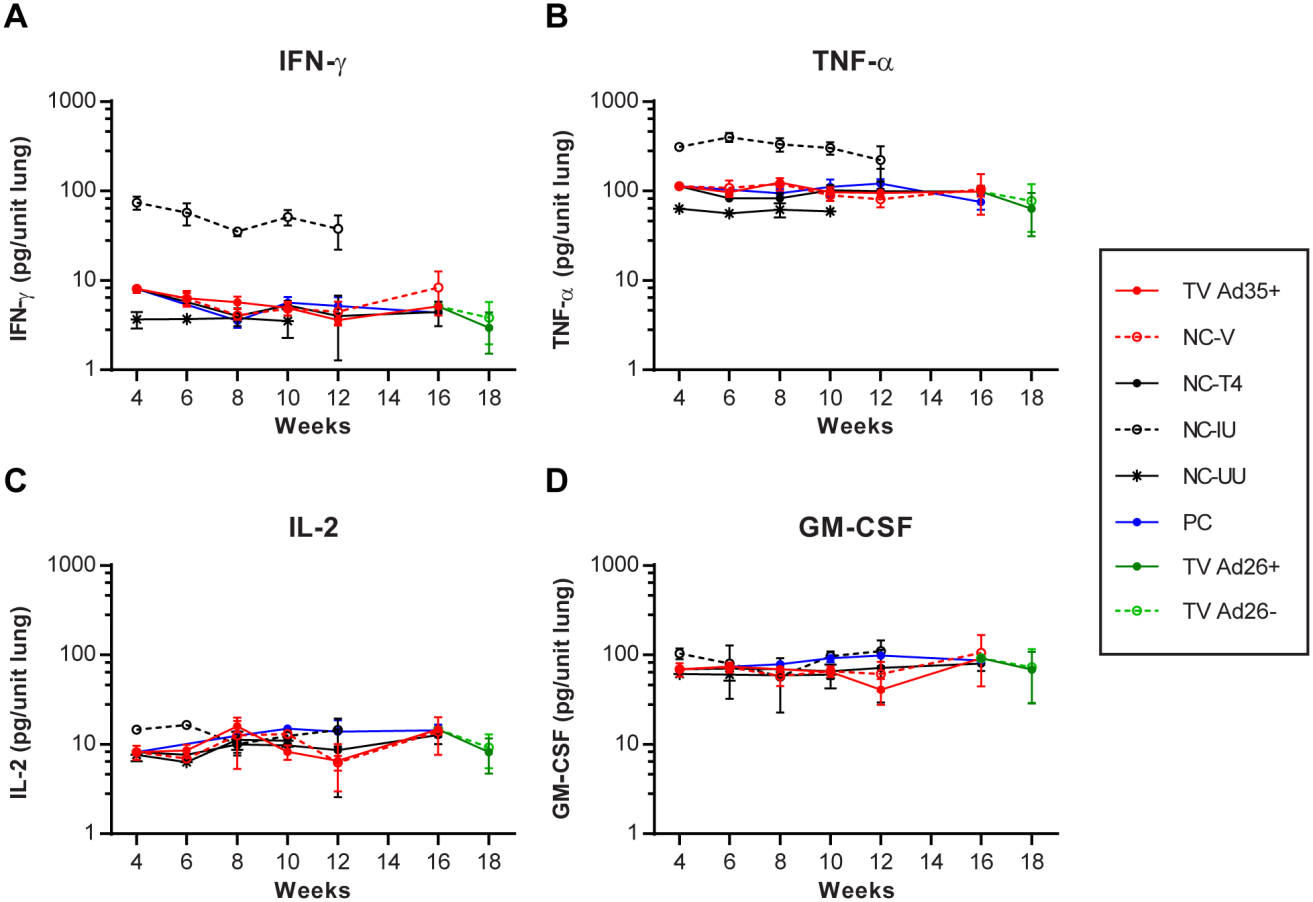
Multiplex enzyme-linked immunosorbent assay of four key cytokines. At each time point 4 weeks post-treatment initiation where lungs were harvested for CFU counting, 150μl of lung homogenate was set aside for cytokine analysis. At week 4, animals in the TV Ad35+, NC-V, NC-T4 and PC groups were identical prior to randomization. Immediately following randomization at week 4, animals in the TV Ad35+ group received immunotherapy with 10^10^ viral particles of Ad35-TBS and animals in the NC-V group received 10^10^ empty Ad35 vector which were both boosted at week 8. At week 16, animals in the TV Ad35+ groups were randomized into TV Ad26+ and TV Ad26- and received 10^10^ viral particles of Ad26-TBS and Ad26 empty vector, respectively. All animals were administered anti-TB therapy except the NC-IU and NC-UU control groups as depicted in Fig 1. Graph depicts the mean cytokine value with standard deviation (n = 4 at each time point for all groups).

In order to study the state of the immune response at the local site of infection, we conducted multiplex enzyme-linked immunosorbent assays to measure the levels of a panel of 23 different cytokines (IL-1a, IL-1b, IL-2, IL-3, IL-4, IL-5, IL-6, IL-9, IL-10, IL-12(p40), IL-12(p70), IL-13, IL-17, Eotaxin, GCSF, GM-CSF, IFNγ, KC, MCP-1, MIP-1a, MIP-1b, RANTES and TNFα) on lung samples obtained throughout the study. Contrary to the splenic ELISpot findings, the multiplex analysis revealed no significant differences in the IFN-γ levels of TV Ad35+ and TV Ad26+ boosted groups compared to controls (NC-V, NC-T4, PC and TV Ad26-; ANOVA) (Fig 5a). On the other hand, the levels of IFN-γ in the infected untreated animals (NC-IU) were visibly higher than all treated groups by week 12 (p<0.0001, ANOVA) (Fig 5a). These findings suggest that IFN-γ levels may be linked closely to the amount of bacteria present in the system rather than a measure of protective or sterilizing immune responses.

We also measured the level of TNF-α, which is a cytokine implicated in macrophage activation and control of intracellular *M*.*tb*. Similar to IFN-γ levels, differences in TNF-α production between animals that underwent treatment were hard to discern. In particular, we observed no obvious differences between the TV Ad35+ group and the control groups and boosting with Ad26-TBS exerted no additional effects on TNF-α secretion (ANOVA) (Fig 5b). Nonetheless, in the case of the infected untreated group of animals NC-IU, significant increases in TNF-α production could be measured compared to all other groups (p<0.0001, ANOVA; week 6) which declined by week 12, when the animals became moribund (p<0.01, ANOVA; week 12) (Fig 5b).

IL-2 is a cytokine that has been implicated in protection against TB and its secretion by T-cells is routinely measured to assess immunogenicity of new TB vaccines in clinical trials. We therefore quantified levels of IL-2 to examine if vaccination would result in the induction of IL-2-producing cells in the lung. Neither immunotherapy with Ad35-TBS or Ad26-TBS was able to augment the production of IL-2 that was clearly detectable in this assay (ANOVA) (Fig 5c). Interestingly, unlike for IFN-γ and TNF-α, the production of IL-2 followed different dynamics in the infected untreated NC-IU animals. Specifically, significant IL-2 production at the beginning of infection (p<0.0001, ANOVA, week 6) began to wane as infection became chronic, when the IL-2 levels became indistinguishable from the treated animals. Of the 20 other cytokines measured in this study, we found a similar trend when we examined the levels of GM-CSF where no obvious differences were seen in the cytokine production between infected untreated and treated animals (ANOVA) (Fig 5d, S3 and S4 Figs, S1 File).

Overall, the multiplex study demonstrated the failure of immunotherapy to augment the production of IFN-γ, TNF-α, IL-2 or other cytokine responses, highlighting a disparity between vaccine-induced immune responses found in the spleen and immune activation in the lungs (Fig 5a–5c, S3 and S4 Figs, S1 File). Furthermore, the contrast between abundant IFN-γ and TNF-α responses and low levels of IL-2 and GM-CSF in the chronically infected animals hint at potential missing components required for effective *M*.*tb* immunity.

### Dynamics of bacterial replication and relapse

**Fig 6:**
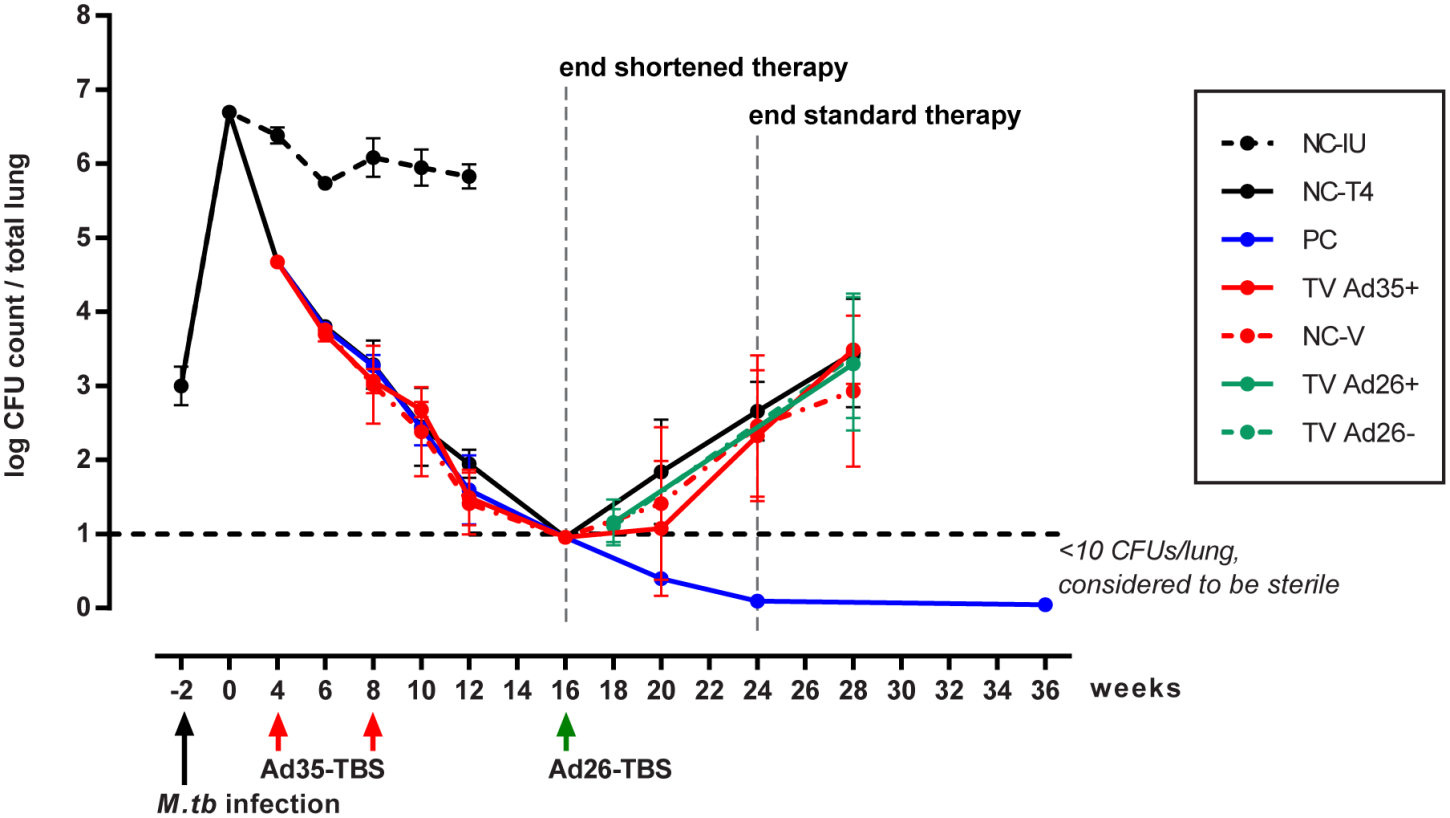
Lung bacterial burden as measured by colony forming units (CFU) over time. At week -2, mice were infected via aerosol with *M*.*tb* at exponential phase of growth. One day following infection, 5 mice from the NC-IU group were sacrificed to determine the infection load. Following CFU enumeration at week 0, mice were randomized and treatment with R, H and Z was initiated for all groups except the NC-IU group as depicted in Fig 1. Mice from the chronically infected group, NC-IU, became progressively moribund and were sacrificed at week 12. At times indicated, immunotherapy with Ad35-TBS and Ad26-TBS were administered via intramuscular injections to the TV Ad35+ and TV Ad26+ animals, respectively. Animals in the NC-V and TV Ad26- received empty Ad35 and Ad26 vectors at the same immunotherapy time points. At week 16, treatment was truncated for mice in the NC-T4, TV Ad35+, NC-V, TV Ad26+ and TV Ad26-. Animals in the PC group received the full treatment through to week 24. CFU enumeration was conducted every 4 weeks following treatment cessation to monitor relapse in all groups that underwent therapy. The lower limit of detection was set at 10 CFU per total lung. Graph depicts the mean CFU value with standard deviation (n = 23, weeks 28 and 36). At all time points measured, no significant statistical differences were noted between the TV Ad35+ group, the vector control group, NC-V, and the main control group, NC-T4 (ANOVA) Boosting with Ad26-TBS at week 16 did not result in decreased bacterial burden by week 28 compared to all other groups in the study (p = 0.14, ANOVA)

To monitor the number of colony forming units (CFU) over time, mice were sacrificed at each time point indicated in Fig 1 and Table 1. On the first day of the experiment (week -2), BALB/c mice were infected via aerosol with an exponential phase culture of *M*.*tb* H37Rv. One day later, mice were sacrificed to determine the bacterial burden at the start of infection and the mean lung CFU counts (+/- SD) were found to be 2.99 +/- 0.26 log_10_. Two weeks later, the number of CFUs increased to 6.69 +/- 0.02 log_10_ by week 0. At this time (designated week 0), animals were divided into groups that received the initiation phase of the treatment with RHZ (PC, NC-T4, TV Ad35+ and NC-V) and those that were left untreated (NC-IU). For the NC-IU animals, the CFU counts plateaued as infection entered the chronic phase and dropped slightly to 5.83 +/- 0.16 log_10_ by week 12 when the animals became moribund and were sacrificed (Fig 6).

Four weeks after treatment was initiated, CFU numbers dropped to 4.68 +/- 0.01 log_10_. Mice were then divided into groups that received vaccination at weeks 4 and 8 (TV Ad35+ and NC-V) and those that did not (PC and NC-T4). At week 8, all groups were switched to the continuation phase of therapy consisting of RH only. By week 16, the CFU count of all groups that received therapy were below the lower limit of detection (LLOD) which was set at 10 CFUs per total lung and therapy was stopped for all groups except the PC group. By this time point, we did not notice any differences in the rate of CFU decrease between the vaccinated and unvaccinated groups of animals (Fig 6).

As expected, the CFU count of animals in the control groups (NC-T4 and NC-V) began to steadily increase following the cessation of treatment (Fig 6). At the final relapse time point at week 28, the CFU counts for the negative control groups NC-T4 and NC-V were 3.44 +/-0.73 log_10_ and 2.93 +/-1.020 log_10_, with 95.7% and 87.5% of animals respectively having relapsed (Fig 6). On the contrary, the positive control group (PC) successfully eliminated the infection by week 36 where none of the animals were found to have relapsed (Fig 6). Given that both the negative and positive controls worked as expected, we were confident of the model’s validity in this experiment.

In contrast to the PC group, all vaccinated groups relapsed indistinguishably from the negative controls despite significant immune responses observed as measured by splenic ELISpot. By week 28, 100% of the TV Ad35+ group relapsed with a CFU count of 3.49 +/-0.46 log_10_ (Fig 6). At all time points measured, no significant statistical differences were noted between the TV Ad35+ group, the vector control group, NC-V, and the main control group, NC-T4 (ANOVA). Boosting with Ad26-TBS when the bacterial count was undetectable at week 16 had no effect in inducing sterilizing immunity in the TV Ad26+ group where 91.3% of the animals relapsed with a CFU count of 3.30 +/- 0.840 at week 28, which was comparable to all other groups at this time point (p = 0.14, ANOVA) (Fig 6).

### Probing the mechanism of efficacy failure

**Fig 7:**
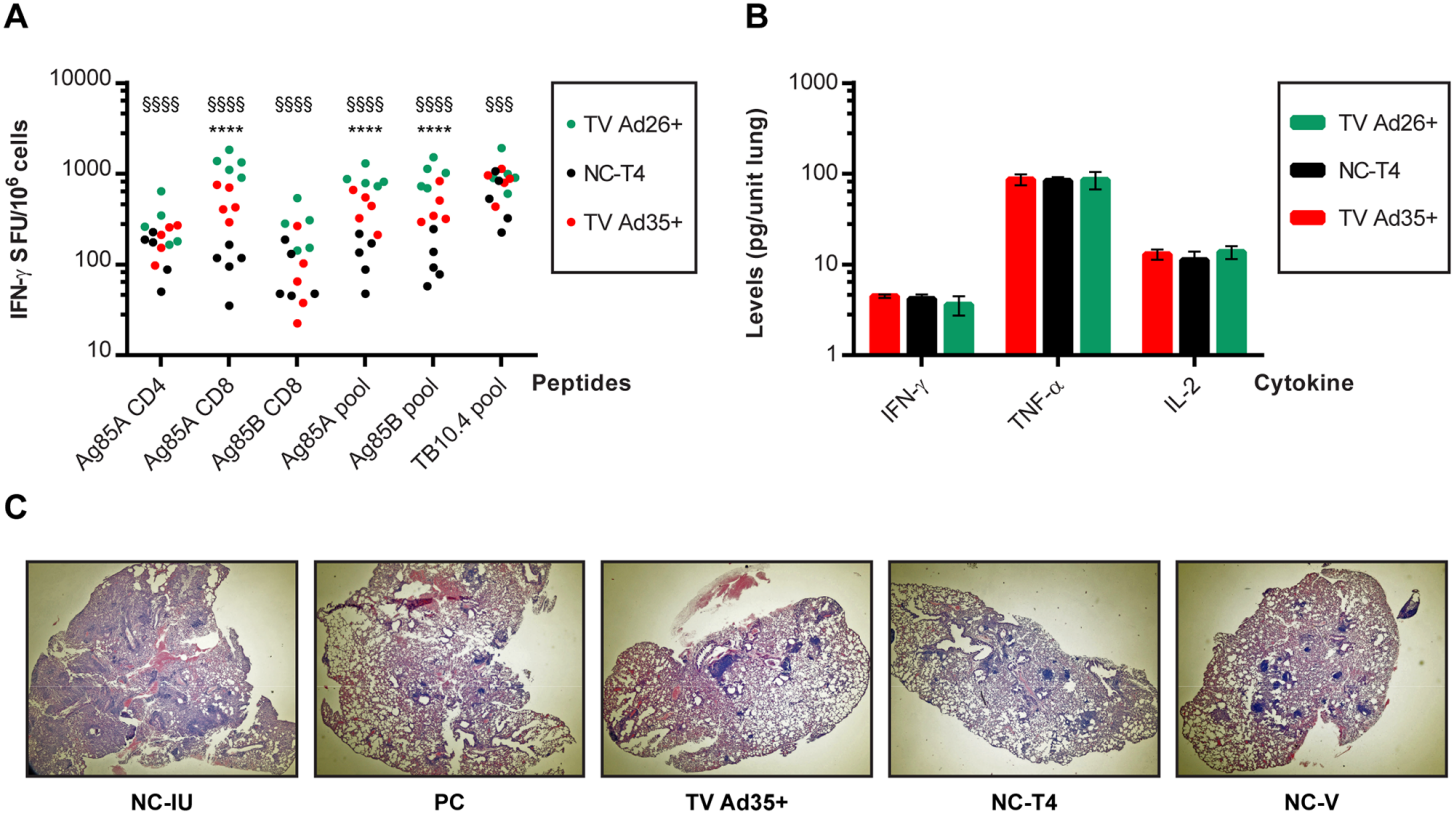
Probing the mechanism of failure at week 28. A) ELISpot analysis was conducted at the final relapse time point of week 28 as previously described. Results of groups that received immunotherapy (TV Ad35+ and TV Ad26+) and the main negative control group NC-T4 are depicted. Results of significance testing are depicted by * for comparison between TV Ad35+ and NC-T4 whereby: * p<0.05, ** p<0.01, *** p<0.001 and **** p<0.0001. § denotes significant differences between TV Ad26+ and NC-T4 whereby: § p<0.05, §§ p<0.01, §§§ p<0.001 and §§§§ p<0.0001. Statistical significance was determined by ANOVA followed by Tukey’s multiple comparisons test where n = 5. B) Multiplex enzyme-linked immunosorbent assay was carried out at week 28 on lung samples as previously described. Results from groups that received immunotherapy (TV Ad35+ and TV Ad26+) and the main control group NC-T4 are shown (mean and standard deviation, n = 4 per group). No statistically significant differences in the levels of IFN-γ, TNF-α or IL-2 were measured between all groups (IFN-γ: p = 0.35; TNF-α: p = 0.98; IL-2: p = 0.09; ANOVA). C) Histopathology of lung samples at week 12 was examined for signs of immunopathology following two doses of immunotherapy with Ad35-TBS. In comparison to the NC-IU animals, the lungs of animals that underwent therapy appeared similar with minimal signs of lesions, necrosis or significant immune infiltration. Immunotherapy did not result in observable immunopathology. Results of histopathology at week 12 are representative of all time points measured (weeks 4, 6, 8 and 10) and at week 18 following Ad26-TBS boosting (data not shown).

We examined the state of the immune system at week 28, to explore possible explanations for the failure of immunotherapy in preventing relapse. Analysis of splenocyte IFN-γ production to pooled peptides revealed significant elevation of immune responses in the immunotherapy groups TV Ad35+ and TV Ad26+ to Ag85A and Ag85B compared to the NC-T4 control but this difference was not seen in antigen TB10.4 responses following Ad35-TBS vaccination but only after Ad26-TBS boosting (Fig 7a). Whilst the levels of Ag85A CD4+ IFN-γ production in the TV Ad35+ and TV Ad26+ animals at week 28 did not defer considerably from the last measurements taken at weeks 16 and 18 respectively, we noticed an increase in the amount of CD4+ T-cells producing IFN-γ in the NC-T4 control group between weeks 10 to 28 (p<0.01, t-test). This ‘catch-up’ response in the control group resulted in no distinguishable differences in CD4 Ag85A IFN-γ values between TV Ad35+ and the control group NC-T4, although boosting with Ad26-TBS did increase the responses further (p<0.0001; ANOVA) (Fig 7a). On the contrary, the level of Ag85A CD8+ IFN-γ production was markedly higher in the TV Ad35+ and TV Ad26+ animals as opposed to the NC-T4 control (TV Ad35+: p<0.0001; TV Ad26+: p<0.0001; ANOVA) (Fig 7a). These results demonstrate that immunotherapy with Ad35-TBS induced both CD4+ and CD8+ responses to Ag85A early in treatment, but only CD8+ responses remained significantly elevated compared to the NC-T4 control by the end of the study. Boosting with Ad26-TBS helped to heighten T-cell responses to all vaccine antigens further by the final time point at week 28.

At the local site of infection, multiplex cytokine analysis of lung samples revealed no significant differences in IFN-γ, TNF-α and IL-2 secretion between TV Ad35+ and TV Ad26+ groups and their respective controls (IFN-γ: p = 0.35; TNF-α: p = 0.98; IL-2: p = 0.09; ANOVA) (Fig 7b). Cytokine levels remained similar to that shown in Fig 5 at earlier time points, as bacterial burden at week 28 (~3 log CFU) did not surpass counts at week 4 (~4.5 log CFU) to show discernible increases in cytokine levels. Crucially, immunotherapy did not increase the generation of these cytokines, suggesting that lack of efficacy may be partly due to failure in inducing lung immune responses.

Finally, failure of efficacy could also be attributed to immunopathology. However, histopathology conducted prior to and after administration of immunotherapy with Ad35-TBS and Ad26-TBS did not reveal any signs of immunopathology at all time points measured (Fig 7c, data not shown). In contrast to lungs of infected untreated animals (NC-IU), lungs of animals that underwent drug therapy, including ones that were immunized, appeared healthy with normal air space and minimal signs of lesions, necrosis or significant immune infiltration (Fig 7c). Hence, immunopathology does not appear to be the mechanism underlying the failure of immunotherapy to show efficacy.

## Discussion

We undertook this study with the simple aim of determining whether immunotherapy with two adenoviral vaccines carrying potent *M*.*tb* antigens could reduce relapse of *M*.*tb* infection when treatment duration was shortened. The results suggest that, in our model, boosting the immune responses during treatment had no effect on reducing bacterial burden or preventing relapse.

### The induction of non-sterilizing T-cell immune responses

Our immunogenicity experiments revealed that immunotherapy with Ad35-TBS and Ad26-TBS induced significant splenocyte IFN-γ responses to all three vaccine antigens Ag85A, Ag85B and TB10.4 when compared to the treated unvaccinated NC-T4 control (Fig 2). Examination of the T cell response to Ag85A revealed that both CD4+ and CD8+ T-cells were induced by immunotherapy (Fig 3). Nonetheless, the magnitude of the CD8+ response was measurably higher by the final time point (Fig 7a). This may not be so surprising, since CD8+ activation following Ad35-TBS vaccination appears to be more prominent in BALBc mice [10]. Thus, it is possible that the usage of mice with diverse genetic backgrounds may reveal a different T cell bias. However, since CD8+ responses have been shown to be important in the control of chronic/latent *M*.*tb* infection, this model should therefore have been advantageous for illustrating any vaccine-mediated effect acting via CD8+ T-cells [19,20]. It was therefore surprising that the heightened CD8+ responses induced by immunotherapy were unable to eliminate persisting bacteria and prevent relapse at week 28 (Fig 6).

Contrary to the CD8+ Ag85A responses, we noticed that the CD4+ Ag85A responses were evidently reduced. Specifically, these CD4+ responses were not significantly different from the responses developed in chronically infected animals (Fig 4A and S2 Fig). The inability of immunotherapy to boost CD4+ IFN-γ levels to Ag85A significantly above the maximum values achieved in chronic infection is intriguing, as it suggests a physiological limitation imposed on the numbers of IFN-γ-producing CD4+ cells at a given moment in time. Recent studies have shown the importance of CD4+ T cell regulation by the inhibitory receptor PD-1, which when absent, resulted in CD4+-mediated immunopathology that exacerbated TB infection [21]. Thus, tight control of CD4+ induction may be a beneficial host regulatory mechanism to prevent immune-mediated pathology. If so, further understanding of the inherent limitations in the immune response and its relation to vaccine-induced immunity is warranted due to its important implications on vaccine development that has been largely focused on CD4+ T cell induction.

### Absence of lung TB immunity

Despite the robust splenocyte IFN-γ production, we cannot exclude that immunotherapy produced T-cells with reduced functional capacity as we did not measure additional parameters such as polyfunctionality or CD8+ cytotoxic potential. It is also possible that intramuscular immunization failed to induce T-cells with the capacity to hone towards the lungs. The finding that there were no evident increases of key cytokines such as IFN-γ, IL-2 and TNF-α in the lungs following immunization as measured in the multiplex assay supports this hypothesis. Although we cannot exclude that the absence of significant changes in cytokine levels was due to limitations in multiplex assay sensitivity, the extreme sensitivity of the assay with a dynamic range of 1,000–20,000 fold and an ability to measure molecules in the range of pg/ml makes this scenario unlikely (S1 File).

Additionally, the mode of vaccine delivery could also determine the efficacy of immunotherapy. Aerosol immunization of AERAS-402/Ad35-TBS has been shown to induce potent and stable effector T-cells in the lungs of rhesus macaques [22]. Although beyond the scope of the current study, immunotherapy administered intranasally or via aerosol followed by further detailing of lung T-cell subtype and function could be subsequently used in our model to determine if therapeutic vaccination that induces lung cellular immunity may have an effect on relapse.

The discrepancy between measurement of immune responses in the spleen and lungs as shown in this study is of significance due to the routine evaluation of new TB vaccines in experimental model systems and clinical trials based on the analysis of peripheral immune responses. Consequently, the results obtained have been shown to bear little correlation to protection against TB [7,23]. In particular, the study by Kagina *et al*. questions the relevance of CD4+, CD8+ and γδ T-cells secreting IFN-γ, TNF-α, IL-2 and IL-17 for TB protection induced by BCG as measured by whole blood obtained from 10-week old infants [23]. Together, these studies highlight the need to develop surrogate markers of protection that reflect the actual state of the immune response at the site of infection.

### Antigens with different immunogenicity profiles

Through our immunogenicity experiments, we observed that not all antigens elicited the same magnitude of immune responses from splenocytes. This was evident when comparing the CD8+ response between Ag85A and Ag85B, implying that caution is needed when generalizing the importance of CD4+ or CD8+ T-cells in the control of TB infection as T-cell contribution may well be antigen specific (Fig 3B and 3C, Fig 7A). Although we demonstrated in our study that CD8+ Ag85A responses were unable to control the on-going infection, it may be possible that other antigens not contained within the vaccine will act protectively via CD8+ or CD4+ T-cells. This leads us to the question on whether the current vaccine antigens are presented efficiently by *M*.*tb*-infected cells in the lungs, resulting in low activation of T-cells at the site of infection. In chronic infection, low levels of Ag85B within granulomas have been shown to adversely affect T-cell activation [18,24]. However, we hypothesize that in our study, downregulation of Ag85B in the lungs by *M*.*tb* may not fully explain the lack of efficacy since immunotherapy with Ad26-TBS at week 16 should have mobilized adaptive immunity two weeks later when rapid bacterial replication resumes, leading to the abundant expression and production of Ag85B. Rather, immunotherapy inefficacy may originate from issues in processing of antigen within the infected cell. This was recently demonstrated in the case of TB10.4, where the primary protein structure was found to be suboptimal for proteosomal processing, compared to a closely related protein, TB10.3, which markedly increased epitope-specific CD8+ responses [25]. Although these high TB10.3 CD8+ responses were unable to protect against TB infection, we cannot exclude that a similar antigen processing issue with Ag85A and Ag85B could be present leading to inferior induction of functionally active T-cells.

### The potential role of IL-2 and GM-CSF in immune protection against TB

The inclusion of the infected untreated control group (NC-IU) allowed us to compare the immune response in chronic infection to the response in animals that were treated and immunized during the early phase of TB therapy simultaneously. It became evident during the study that high IFN-γ production, as observed in the lungs and spleen of animals from the NC-IU cohort did not reflect the presence of protective immune responses (Figs 4 and 5). Furthermore, we noted that as the animals relapsed, the levels of IFN-γ in the unvaccinated treated control animals (NC-T4) also raised (compare TB10.4 pool peptide levels at week 10 in Fig 2C to the final response at week 28 in Fig 7A), a common trend for all antigens tested. Thus, our results corroborate other published observations that IFN-γ in fact correlates with bacterial burden and may not be a good marker for protection [8,26].

Despite the generally raised levels of most lung cytokines measured by the multiplex assay in the chronically infected animals, two cytokines, IL-2 and GM-CSF, stood out in our analysis due to the absence of observable differences between the infected animals and the treated controls (Fig 5). IL-2 is considered to have a crucial function in the programming of T-cells with improved memory capacity and was subsequently shown to be important for protection against TB [27,28]. Mice that are deficient in GM-CSF are unable to contain *M*.*tb* growth and succumb rapidly following infection [29]. Of importance to our study is the recent discovery that GM-CSF produced by invariant natural killer cells (iNKT) is necessary and sufficient to control *M*.*tb* growth [30]. Consequently, the inability to heighten the levels of these cytokines in chronically infected animals may be a hint at the immune factors lacking in the production of sterilizing immune responses and could serve as future biomarkers for monitoring TB immunity in place of IFN-γ.

### Challenges in developing a clinically-relevant model

Previous studies on immunotherapeutic or post-exposure vaccinations have demonstrated efficacy in mice and non-human primates using experimental setups involving incomplete chemotherapy with 2 drugs provided via drinking water or with meals over a period ranging from 6 weeks to 6 months [31–33]. In our study, we aimed to further improve on these models and recapitulate the treatment scheme in humans in order to come up with a model that is as clinically-relevant as possible. Despite this, the relapse model we presented does not completely mimic the situation in humans where 7% of patients relapse compared to above 90% of mice in our study when treatment was shortened to 4 months [34]. The initial challenge dose used in our study, which is routinely administered in the field, may not reflect the reality of *M*.*tb* infection in humans where the initial infection is initiated by far fewer bacilli. Consequently, a high initial challenge dose may result in higher numbers of unculturable persisters at the end of therapy at 4 months, resulting in a high relapse rate that masks the true efficacy of a vaccine. The issue of challenge-dose dependency compromising vaccine or drug efficacy has been demonstrated in the HIV field and therefore is a relevant consideration in the development of TB vaccine model systems [35].

Nonetheless, the permissive tendency of TB mouse model systems to demonstrate vaccine efficacy which is not reproducible in humans due to the development of strong cell-mediated immunity however suggests that a more, rather than less stringent mouse model system needs to be developed. To this end, our model could therefore be a starting point for comparing the efficacy of various other post-exposure vaccines that have demonstrated efficacy in other model systems [31–33,36]. Improvements on the model, including the use of diverse experimental animals and reduction in the relapse rate to mimic that in humans may subsequently be called for to enhance the model’s utility for the identification of efficacious novel TB vaccines.

### The ambiguous location of persister cells

The underlying assumption that formed the basis of our study hypothesis was the concept that phenotypically resistant bacteria persisted intracellularly within host cells. However, the viewpoints of Grosset and Orme regarding the importance of extracellular persisters as part of a more complex *M*.*tb* life cycle deserves further contemplation as it provides an alternative explanation on the failure of cell-mediated immunity to eliminate bacteria in our model [37,38]. Although extracellular bacteria have been mostly observed in guinea pigs and humans where necrosis is predominant, they have also been seen in the lungs of mice that are more susceptible to *M*.*tb* [39]. Furthermore, mild degrees of necrosis are present even in resistant C57BL/6 mice although no acid fast bacilli were detected at these sites by conventional staining methods [39]. It might subsequently be informative to further investigate if the intensity of therapy used in this model and in humans influences the location and physiological state of intracellular persisters that could account for the current requirement for prolonged therapy of 6 months and determine if the failure of our vaccine to prevent relapse is related to *M*.*tb*’s physiological state within the host.

### Concluding remarks

*M*.*tb* is an ancient organism that has evolved a complex life cycle in order to facilitate its own survival and transmission. As such, solutions that do not take into account this complexity will not likely succeed. The notion that hyperconserved T-cell epitopes in the *M*.*tb* genome may actually be promoting immune responses that are beneficial to *M*.*tb* rather than protective to the host exemplifies the need to re-examine the rationale by which antigens are selected for incorporation into vaccines currently in development [40]. Likewise, gaining a deeper understanding into the form and location of persisters in the context of *M*.*tb*’s developmental biology should be prioritized. The results obtained from our study thus emphasize the need to incorporate these multiple perspectives into the design of future vaccines that can effectively target persisters, shorten TB therapy and ultimately eradicate latent TB.

## Materials and Method

### Ethics Statement

All animal work was conducted as per the protocol approved by the Johns Hopkins University Institutional Animal Care and Use Committee (protocol # MO09M101).

### Bacterial Strain and *in vitro* Growth Conditions

*Mycobacterium tuberculosis*, strain H37Rv was used as the infecting strain and grown under standard conditions, in Middlebrook 7H9 media (Difco), supplemented with 0.5% glycerol, 10% oleic acid-albumin-dextrose-catalase (OADC) and 0.05% Tween 80, with constant shaking at 37°C. Mouse organ homogenates were plated on Middlebrook 7H11 selective plates (Becton Dickinson) at appropriate dilutions and CFU were counted after incubation for 4 weeks at 37°C.

### Infection, Immunization and Treatment of Experimental Mice

BALB/c mice, female, 4–5 weeks old (Charles River Laboratories) were housed at Johns Hopkins University in a bio-safety level 3 vivarium. Appropriate nutrition, health monitoring and clinical care were provided throughout the study. Infection of all mice was done at once via aerosol route in a Glas-Col Middlebrook Inhalation Exposure System (Glas-Col Inc) using 10 ml of *M*. *tb* culture at exponential phase of growth, diluted to A_260nm_ of 0.1. In infection cycle of 15 minutes warm-up, 30 minutes aerosolization of *M*. *tb*, 30 minutes of aerosol decay and 15 minutes of decontamination was used. Treatment of mice was initiated at 2 weeks after infection. This time point is referred to as week 0. Treatment included daily (weekdays) oral gavage with isoniazid at 25 mg/kg, rifampicin at 10 mg/kg and pyrazinamide at 150 mg/kg of body weight. Immunizations were performed by intramuscular injection of 1x10^10^ VP in 50 ul aliquots by in each hind leg. Mice were sacrificed to determine bacterial burden at desired time points. The number of mice, their corresponding group and the time points studied are shown in Fig 1 and Table 1. Blood was obtained by cardiac puncture and used for neutralization assays to confirm vaccine uptake (data not shown). Lungs and spleens were aseptically removed, and mechanically homogenized in 0.8 ml of sterile 1X PBS using 2 mm glass beads and a mini beadbeater (BioSpec Products). Organ homogenates were plated on Middlebrook 7H11 selective plates (Becton Dickinson) at appropriate dilutions and CFU were counted after incubation for 4 weeks at 37°C. Lower lobe sections of lungs from each group were obtained, fixed in 10% formalin, and stained with H&E for histopathological studies.

### Splenocyte Isolation and Enumeration

Spleens were aseptically obtained from immunized and control mice, macerated using a sterile tissue culture filter (BD Biosciences, San Jose, CA), and the resulting cell suspension was diluted in Hanks Balanced Salts Solution (Invitrogen, Carlsbad, CA) containing 10% Heat Inactivated Fetal Calf Serum (Invitrogen, Carlsbad, CA). Contaminating red blood cells were lysed using the Mouse Erythrocyte Lysing Kit (R&D Systems, Minneapolis, MN) according to the manufacturer’s directions. Cells were then harvested by centrifugation and diluted into R10 media (RPMI media (Invitrogen, Carlsbad, CA), supplemented with 10% Heat Inactivated Fetal Calf Serum (Invitrogen, Carlsbad, CA), 10 mM HEPES (Invitrogen, Carlsbad, CA), 50 μM 2-mercaptoethanol (Invitrogen, Carlsbad, CA), and 100 μg/mL each of Penicillin G and Streptomycin (Invitrogen, Carlsbad, CA). Cell viability counts were performed on remaining splenocytes using trypan blue exclusion staining with the aid of a light microscope (Fisher Micromaster Inverted, Fisher Scientific, Pittsburgh, PA) and an Improved Nebauer hemacytometer (Hausser Scientific, Horsham, PA). Cells were then diluted using R10 to concentrations ranging from 0.64–4.0 x 10^6^ viable cells/mL.

### ELISpot

Commercially available ELISpot kits were used to measure IFN- γ responses (ELISpot-Plus, MABTECH, Cincinnati, OH) essentially according to the manufacturer’s supplied instructions. Briefly, pre-coated plates were washed with Dulbecco’s Phosphate Buffered Saline (PBS, Invitrogen, Carlsbad, CA), and blocked using R10 media. Plates were then washed and inoculated in duplicate with either R10 media, PMA to serve as positive control or one of the antigens listed in S1 Table to a final concentration of 1 μg/mL. T-cell specific peptides used for in vitro stimulation were purchased from Pepscan Systems (Lelystad, The Netherlands). Pooled peptides covering the complete sequence of Ag85A, Ag85B and TB10.4 (15-mer peptides overlapping by 11 amino acids) were provided by AERAS (Rockville, Maryland). Splenocytes were seeded in each well at 200,000, 80,000 or 32,000 viable cells per well. Plates were sealed and incubated in a humidified CO_2_ incubator (Thermo Scientific) at 37°C. 12–16 hours later, plates were washed and incubated with the supplied biotinylated anti-IFN-γ antibody (R46A2). Plates were then washed and incubated with an alkaline phosphatase conjugated strepdavidin detection reagent. Finally, plates were washed and developed using the NBT/BCIP chromogenic substrate and allowed to develop. Developed plates were washed in water, allowed to dry overnight, and read using a commercially available ELISpot plate reader system (CTL Immunospot 4.0, CTL, Shaker Heights, OH). Data from each well was analyzed in Microsoft Excel, exported into PRISM software (Graphpad Inc, La Jolla, CA) and presented as mean spot forming units per million cells following log transformation.

### Cytokine Multiplex Assay

For each lung sample, 150 ul of homogenate was diluted 1:1 with PBS and filtered through 0.2 um filter (Millipore). The lung homogenate filtrate assayed to determine the levels of cytokines IL-1a, IL-1b, IL-2, IL-3, IL-4, IL-5, IL-6, IL-9, IL-10, IL-12(p40), IL-12(p70), IL-13, IL-17, Eotaxin, GCSF, GMCSF, IFNγ, KC, MCP-1, MIP-1a, MIP-1b, RANTES and TNFα using Bio-Plex Pro Mouse Cytokine 23-Plex Immunoassay kit (BioRad) as per the manufacturers protocol.

### Recombinant Ad35-TBS (AERAS-402) and Ad26-TBS

The construction of a recombinant Ad35 vector expressing Ag85A, Ag85B and TB10.4 have been previously described in detail [10,41]. For Ad26-TBS, the *M*.*tb* antigens Ag85A, Ag85B and TB10.4 were fused directly, resulting in the expression of a single polyprotein as described in [41]. The fragment encoding for these antigens was cloned into the pAdApt26 plasmid which has been described earlier using the HindIII/XbaI sites [42].

### Statistical analysis

Splenocyte and lung CFU counts (y) were log-transformed as log_10_(y) before analysis. Statistical analysis was performed using one-way ANOVA followed by Tukey’s multiple comparisons post-test (GraphPad Prism v.6, GraphPad Software, San Diego, CA) to adjust for multiple comparisons, where appropriate. When only two groups were analysed, student’s unpaired t-test was conducted. Differences were considered significant when the *p*-value was <0.05.

## Supporting Information

S1 Fig ELISpot IFN- γ responses for all groups measured in the study. (TIF)

S2 Fig T-cell responses between animals receiving immunotherapy and chronically infected untreated animals in a preliminary experiment. **A) Lines** depict geometric mean and p values were calculated using t-test (n = 5 at each time point for both groups) whereby; * p<0.05, ** p<0.01, *** p<0.001, **** p<0.0001. SFU = splenocyte forming units.
(TIF)

S3 Fig Multiplex enzyme-linked immunosorbent assay of IL-1α, IL-1β, IL-3, IL-6, IL-9, IL-10, IL-12p40, IL-12p70, IL-13 and KC. **Graph** depicts mean and standard deviation (n = 4 at each time point).
(TIF)

S4 Fig Multiplex enzyme-linked immunosorbent assay of MCP-1, MCP-1α, MCP-1β,RANTES, IL-4, IL-5, GCSF, IL-17 and Eotaxin. Graph depicts mean and standard deviation (n = 4 at each time point).
(TIF)

S1 File Source data for multiple enzyme-linked immunosorbent assay for all cytokines measured. (XLSX)

S1 Table Peptides used in ELISpot analysis. For stimulation of splenocytes, 1μg/peptide/ml was used. aa = amino acid.
(DOCX)
