# Supplementary figures and images for: Immunogenicity without Efficacy of an Adenoviral Tuberculosis Vaccine in a Stringent Mouse Model for Immunotherapy during Treatment
Source: PLoS One. 2015 May 21;10(5):e0127907. doi: 10.1371/journal.pone.0127907 (PMC4440646; doi:10.1371/journal.pone.0127907)

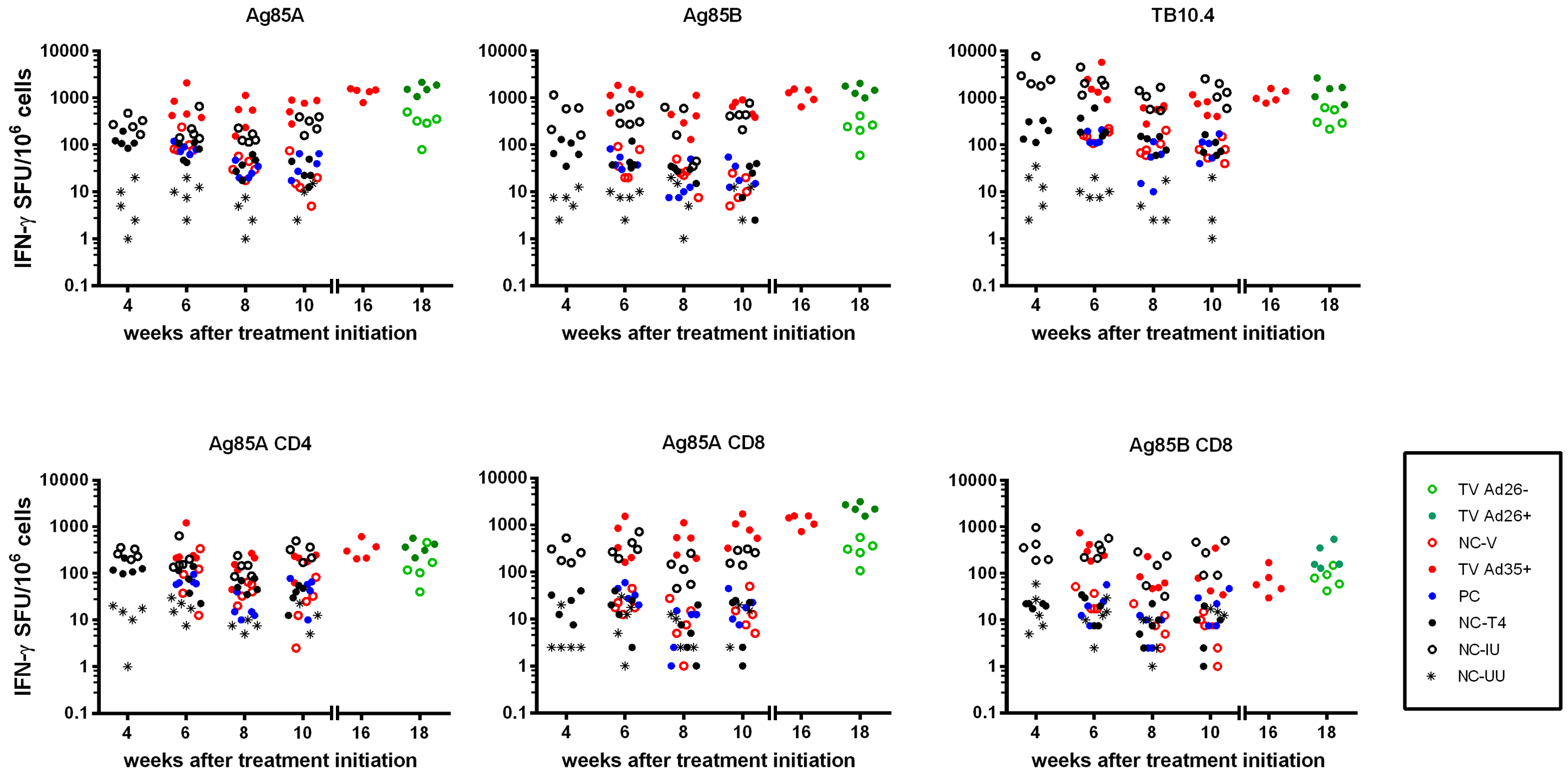

Supplement: S1 Fig — (TIF) [file pone.0127907.s001.tif]

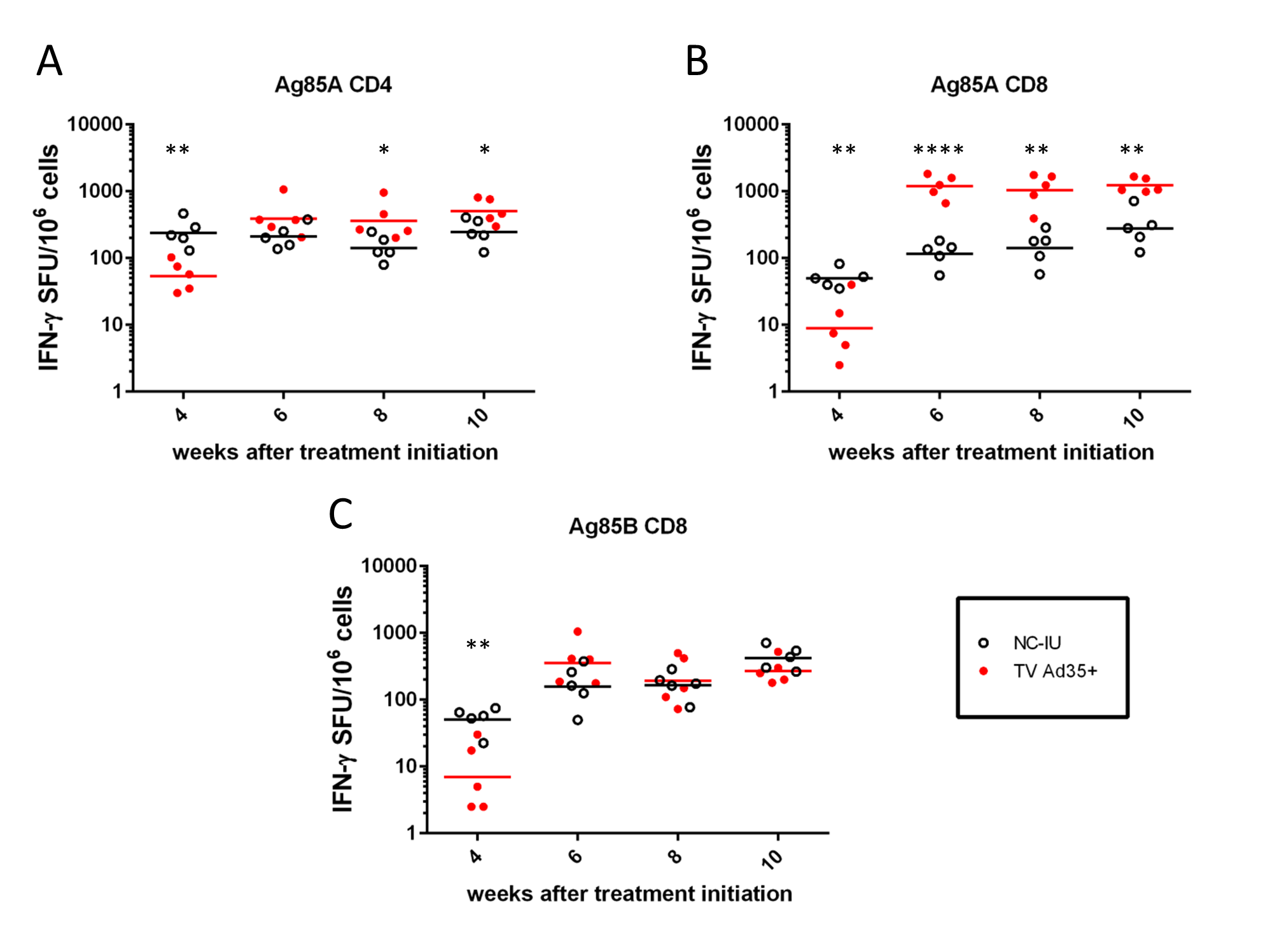

Supplement: S2 Fig — A) Lines depict geometric mean and p values were calculated using t-test (n = 5 at each time point for both groups) whereby; * p<0.05, ** p<0.01, *** p<0.001, **** p<0.0001. SFU = splenocyte forming units. (TIF) [file pone.0127907.s002.tif]

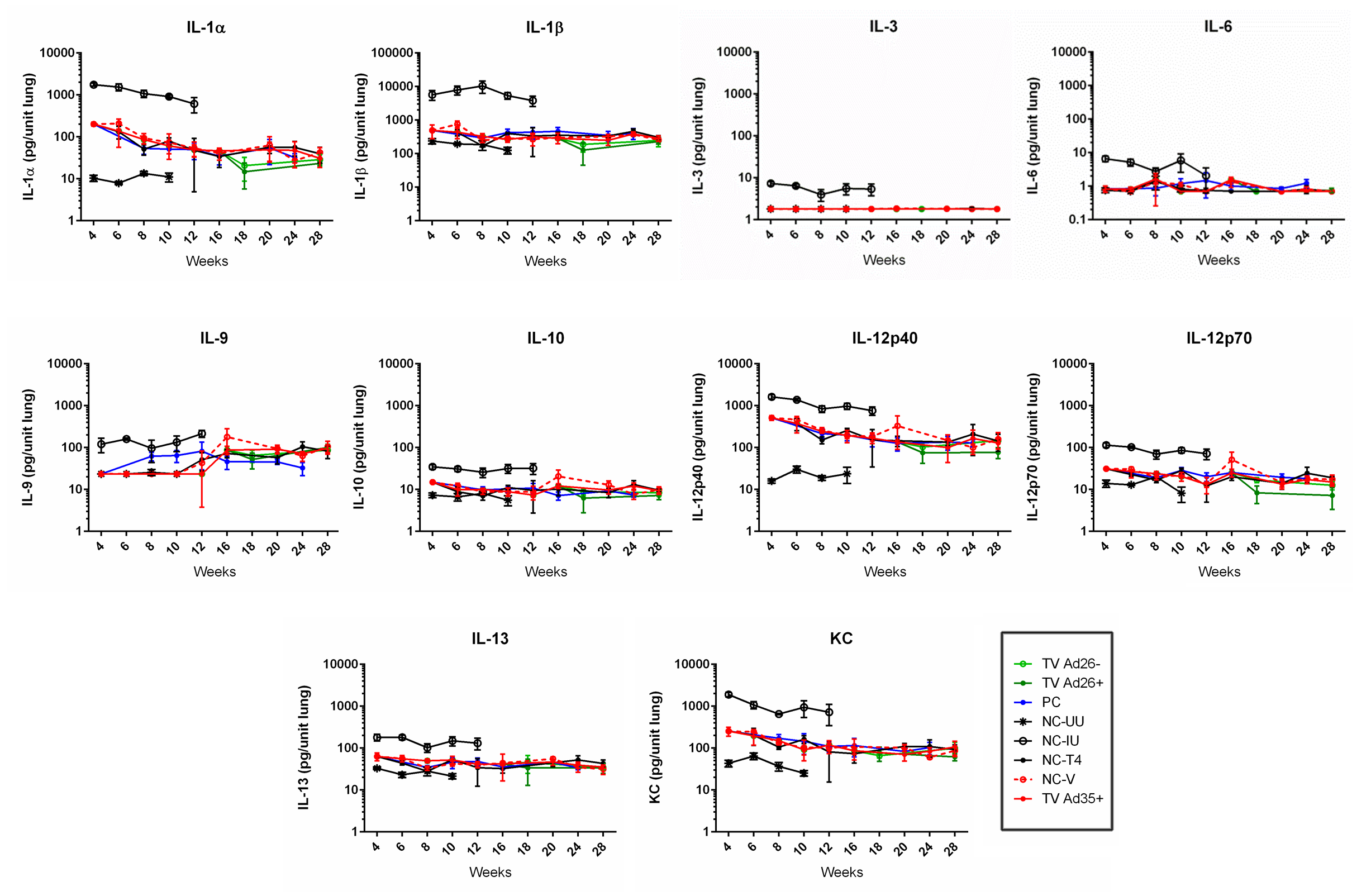

Supplement: S3 Fig — Graph depicts mean and standard deviation (n = 4 at each time point). (TIF) [file pone.0127907.s003.tif]

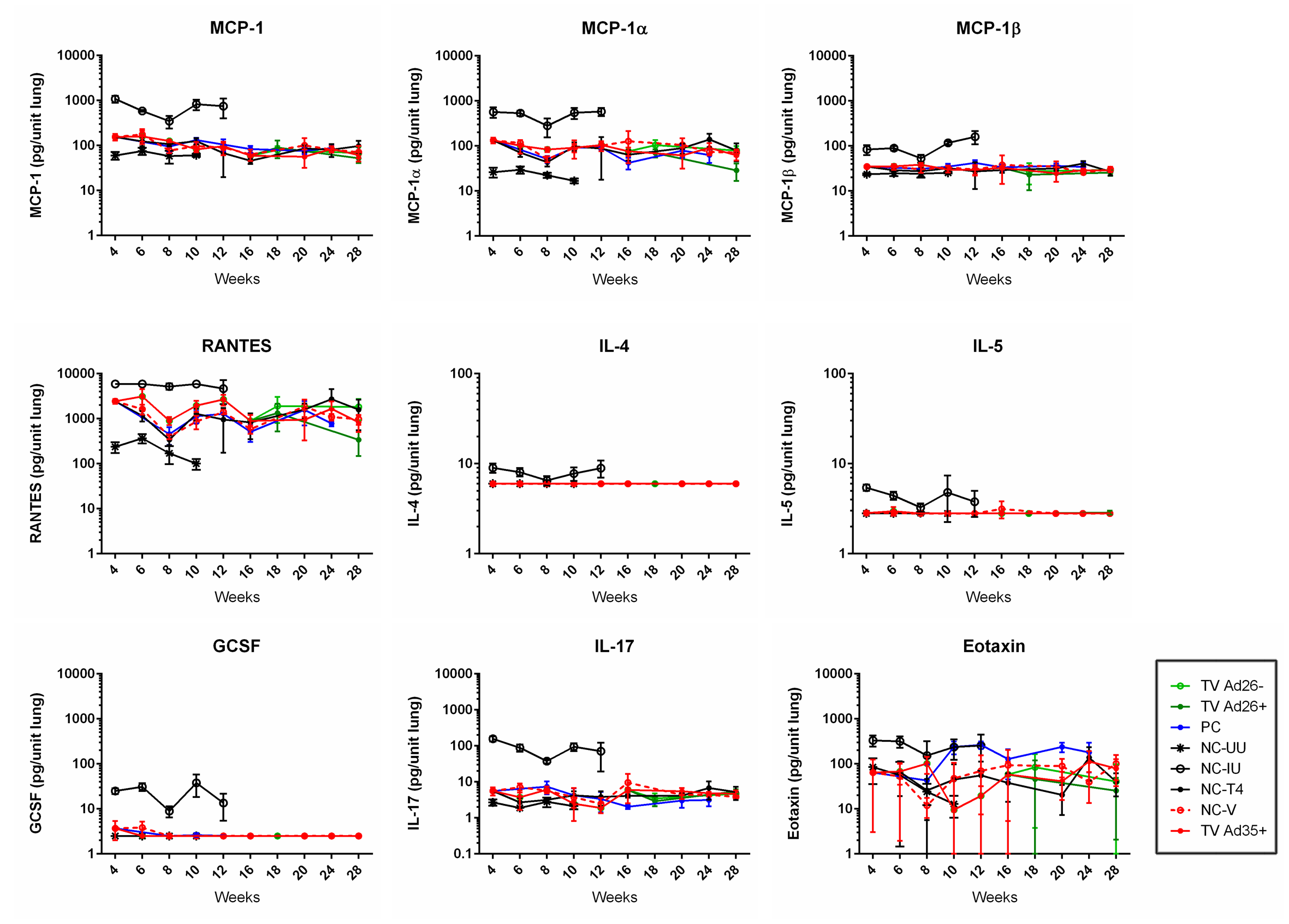

Supplement: S4 Fig — Graph depicts mean and standard deviation (n = 4 at each time point). (TIF) [file pone.0127907.s004.tif]
