# Supplementary material for: Immunogenicity without Efficacy of an Adenoviral Tuberculosis Vaccine in a Stringent Mouse Model for Immunotherapy during Treatment
Source: PLoS One. 2015 May 21;10(5):e0127907. doi: 10.1371/journal.pone.0127907 (PMC4440646; doi:10.1371/journal.pone.0127907)
Supplement: S1 Table — For stimulation of splenocytes, 1μg/peptide/ml was used. aa = amino acid. (DOCX) [file pone.0127907.s006.docx]

S1 Table. Peptides used in ELISpot analysis.

| **Stimulant** | **Peptide sequence** | **Epitope type** |
| --- | --- | --- |
| Ag85A | LTSELPGWLQANRHVKPTGS | CD4 |
| Ag85A | MPVGGQSSF + VYAGAMSGL | CD8 |
| Ag85B | ALLDPSQGMGPSLIG + IYAGSLSAL | CD8 |
| Ag85A | Pool, 15 aa with 11 aa overlap | Mixed |
| Ag85B | Pool, 15 aa with 11 aa overlap | Mixed |
| TB10.4 | Pool, 15 aa with 11 aa overlap | Mixed |

For stimulation of splenocytes, 1µg/peptide/ml was used. aa= amino acid.
